# Comparison of Fluid Analysis and Cytologic Findings of Cerebrospinal Fluid Between Three Collection Sites in Adult Equids With Neurological Disease

**DOI:** 10.3389/fvets.2022.821815

**Published:** 2022-04-26

**Authors:** Kimberly A. S. Young, Kate L. Hepworth-Warren, Katarzyna A. Dembek

**Affiliations:** Department of Clinical Sciences, North Carolina State University, Raleigh, NC, United States

**Keywords:** equine protozoal myeloencephalitis (EPM), cytopathology, atlanto-occipital, atlantoaxial, lumbosacral

## Abstract

Cerebrospinal fluid (CSF) is routinely collected from three sites in the horse, the atlanto-occipital (AO), atlantoaxial (AA), and lumbosacral (LS) space. A comparison between fluid analysis parameters [total protein, total nucleated cell count (TNCC), red blood cell (RBC) count, and morphologic analysis] from samples obtained at each of the three sites has not previously been performed. A retrospective analysis was performed to evaluate the differences in fluid analysis of CSF between the AO, AA, and LS sites in equids presented to a referral service for evaluation of suspected neurological disease. A total of 113 equids aged ≥1 year that underwent CSF collection between 2008 and 2020 were included. Total nucleated cell count, RBC concentration, total protein (TP), and morphologic evaluation between CSF samples obtained from the three sites were compared. When comparing all samples, LS centesis was associated with higher RBC compared to other sites (*p* < 0.05); TP was lower in the AA group than in the LS group (*p* < 0.05). Within a subset of cytologically unremarkable samples, RBC concentration was highest in LS samples (*p* < 0.01); TP was higher in LS samples compared to AA samples (*p* < 0.05) and TNCC was higher (*p* < 0.01) in AA and LS groups compared to the AO. In cytologically abnormal samples, there were no significant differences between sites in any parameter. Abnormal cytology was correlated with non-survival (*p* = 0.0002). Non-survival was associated with higher TNCC (*p* < 0.01). The receiver operating characteristic (ROC) curve for TNCC had an area under the curve of 0.67 (95% CI, 0.55–0.79) and indicated that a cutoff value of 24 cells/μL maximized specificity (72%) and sensitivity (54%) to predict non-survival in all horses. Positive predictive value was 45%; negative predictive value was 78%. The concentration of RBC was higher in samples from the LS site. This has clinical implications due to the importance of comparative diagnostics and its potential impact on cytologic evaluation. There were minimal differences in multiple other parameters between sites, which are likely clinically insignificant.

## Introduction

Equine neurological disease often presents a diagnostic challenge to clinicians due to the size of the patient limiting access to advanced imaging techniques and the inherent risk to both the patient and clinician during collection of cerebrospinal fluid (CSF) (1). While CSF is frequently used for infectious disease testing, cytologic evaluation of the fluid provides additional insight into disease processes and physiologic status, including the integrity of the blood–brain barrier (2). Multiple sites have been described for CSF collection in the horse including the atlanto-occipital (AO) space (3), atlantoaxial (AA) space (4), and lumbosacral (LS) space (1). Centesis of the AA and LS sites is typically performed under standing sedation, while AO centesis is most frequently performed with the patient in lateral recumbency under general anesthesia, though a standing technique with ultrasound guidance has been described (5).

Site selection for CSF collection is initially guided by neurolocalization. A site near or just caudal to the region of interest identified by neurolocalization is often suggested as the appropriate site for sampling (1). Due to the fact that many common causes of neurological disease in the horse are either multifocal [e.g., equine protozoal myeloencephalitis (EPM)], diffuse (e.g., equine herpes myeloencephalopathy), or localized to the cervical spinal cord (e.g., cervical vertebral stenotic myelopathy), centesis sites near the brain are often preferred (6). Beyond neurolocalization, site selection is influenced by the desire to minimize blood contamination that could falsely increase total protein (TP), total nucleated cell count (TNCC), or antibodies within a sample, and by the effect of the patient's ataxia and mentation on the ability to be sedated and remain standing for a procedure (7). In severely ataxic patients, recovery from general anesthesia can be difficult; thus, standing techniques are often preferred. While the LS site is still a widely used location, the technique described by Pease et al. in 2012 to access the AA space has become increasingly popular due to its technical ease, lack of reaction from patients, safety for the practitioner, and since it lacks the requirement for general anesthesia (8, 9).

Once collected, CSF is often submitted for bacterial culture, antibody titers, biomarker evaluation, or fluid analysis and cytology. Parameters for fluid analysis are often applied universally independent of site of collection. However, in humans, CSF obtained from different sites is significantly different, with protein in samples obtained from lumbar puncture being 1.6 times greater than that of samples obtained from the ventricles (10). IgG and albumin in CSF from lumbar puncture in humans are more than double the immunoglobulin G (IgG) and albumin in ventricular CSF (10). Similar trends have been documented in both healthy and diseased dogs wherein TP and TNCC were both higher in more caudal sites (11, 12). In a group of 45 neurologically normal horses and ponies, CSF obtained from the LS space and the AO space was not significantly different in TNCC or TP concentration, although glucose was higher in LS samples (13). The subarachnoid space accessed between the first and second cervical vertebrae (AA space) is anatomically separate from the cerebromedullary cistern accessed through AO puncture (4). Recent literature has shown no difference in nucleated cell count in CSF from horses with no evidence of neurological disease when sampling the AA and the AO sites, but did show higher TP in samples from the AA site (14). While CSF collected from the AA and LS sites (8) and CSF from the AO and AA sites have previously been compared (14), there are no published comparisons between all three collection sites. Separate reference ranges for albumin have been proposed in human literature based on sample site, but specific reference ranges for CSF collected from the AA site in horses have not yet been established (10). The objective of the current study was to compare RBC concentration (indicative of blood contamination, or in less frequent cases, hemorrhage), TNCC, and protein concentrations between CSF collected via AO, AA, and LS centesis from horses that were presented for evaluation of suspected neurological disease over a 12-year period. The authors hypothesized that there would be significant differences in these values between the three sites in horses, regardless of neurological disease status, that would support the establishment of separate reference ranges for use in evaluation of CSF from the AA site. A secondary objective was to evaluate non-survival and association with fluid analysis parameters. The authors hypothesized that elevated TNCC (pleocytosis) would be associated with non-survival.

## Materials and Methods

An electronic medical record search was performed to identify all horses 1 year or older that were presented to the North Carolina State University Veterinary Teaching Hospital from 2008 until 2020 and underwent antemortem CSF collection with cytologic evaluation and fluid analysis. Exclusion criteria included samples that were collected post-mortem, samples without fluid analysis or cytologic evaluation, and samples obtained from patients less than 1 year of age as foals have been shown to have higher TP in CSF than adult horses (15). Data extracted from each record included age, breed, gender, results of quantified fluid analysis (TNCC, RBC, and TP), results of cytologic evaluation, survival, final diagnosis (if present), presenting complaint, and any concurrent diagnoses noted in record. Neurological examination findings were noted when reported. Though most of the subjects were horses, donkeys were not excluded as they have been previously established to have similar CSF composition to horses (16). Samples were evaluated both objectively (TNCC, RBC concentration, and TP) and subjectively via microscopy by a board-certified clinical pathologist. Analysis in the diagnostic lab includes gross analysis of fluid, cytospins in bovine albumin, manual count with hemocytometer, and morphologic evaluation. Protein evaluation is performed on a chemistry analyzer (Cobas c501).

### Data Classification and Grouping

Subjects were divided by collection site into AO, AA, and LS groups and TNCC, RBC concentration, and TP were compared between these three groups. The same parameters were also compared between sites in horses classified as neurologically abnormal or neurologically normal, between survivors and non-survivors, between sites in samples classified as cytologically remarkable (abnormal) and unremarkable, and between sites in samples classified as blood admixed (diluted by peripheral blood) or those not considered to be blood admixed (not blood admixed).

#### Qualification of Samples as Cytologically Unremarkable or Abnormal

Samples were classified as cytologically unremarkable or abnormal based on wording of official pathology reports from board-certified clinical pathologists. Reports that did not specifically state “cytologically unremarkable” or “abnormal” were reviewed by a second board-certified clinical pathologist prior to classification. Samples with RBC concentration and TNCC within laboratory-established reference ranges and no clinically significant morphologic changes were classified as cytologically unremarkable. Abnormal samples were those that had significant abnormalities either via cell count or microscopic evaluation, though blood admixture alone was not considered sufficient to classify a sample as abnormal.

#### Qualification of Blood Admixed Samples

To investigate the effect of higher red blood cell count (blood admixture) on TNCC and TP, two analyses were performed to compare blood admixed samples and non-blood admixed samples. The first used subjective classification from the pathology report to classify all samples with the words “hemodilute” or “hemodilution” as blood admixed. The second used a cutoff of ≥500 RBC/μL to classify samples as blood admixed, consistent with studies in other species (17, 18).

### Statistical Analysis

Data were tested for normality by a Shapiro–Wilk test and were not normally distributed. Median and ranges were calculated for continuous variables. Kruskal–Wallis statistics with Dunn's *post-hoc* test was used to compare three groups and Mann–Whitney test was used to compare two groups. Relationships between survival and categorical variables were analyzed using contingency tables and Fisher's exact test. To determine the area under the curve (AUC) and a cutoff value above which non-survival could be most reliably predicted by TNCC, a receiver operating characteristic (ROC) curve was calculated. Significance was set at *p* ≤ 0.05.

## Results

### Patient Demographics

A total of 113 records were included in the final analysis, including 36 mares, 72 geldings, and 5 intact males. Median age was 11 years old, and range was from 1 to 30 years old. Breeds represented included the following: Warmblood (*n* = 27), Quarter Horse (*n* = 27), Thoroughbred (*n* = 17), Tennessee Walking Horse (*n* = 8), Paint Horse (*n* = 5), Pony (*n* = 4), Arabian (*n* = 4), Friesian (*n* = 3), Morgan (*n* = 3), Saddlebred (*n* = 2), Standardbred (*n* = 2), Irish Sport Horse (*n* = 2), and Appaloosa, Appendix Quarter Horse, Clydesdale, Fox Trotter, Gypsy Vanner Horse, Haflinger, Paso Fino, Percheron, and miniature donkey (1 each). All patients were evaluated at the NCSU Veterinary Teaching Hospital for suspected neurological disease. Presumed or confirmed neurological diagnoses included EPM (*n* = 21), cervical vertebral stenotic myelopathy (*n* = 12), osteoarthritis of vertebral facets (*n* = 12), Eastern Equine Encephalitis (*n* = 3), botulism (*n* = 3), traumatic skull fracture (*n* = 3), polyneuritis equi (*n* = 2), neuroborreliosis (*n* = 2), neoplasia (*n* = 2), and epidural hematoma, sphenopalatine mass, shivers, temporohyoid osteoarthropathy, facial neuritis, atlantoaxial malformation, retrobulbar mass, epilepsy, narcolepsy, malnutrition resulting in seizures, cervical myelitis, Actinobacillus encephalitis, and stringhalt (1 each). Eight patients had multiple neurological diagnoses and 12 had concurrent non-neurological disease. Two patients were diagnosed with colic, 4 had orthopedic disease, and 1 each was diagnosed with equine multinodular pulmonary fibrosis, polysaccharide storage myopathy, pituitary pars intermedia dysfunction, immune-mediated keratoconjunctivitis, equine recurrent uveitis, delayed hypoglobulinemia, ethmoid hematoma, aspiration pneumonia, tooth root abscessation, equine gastric ulcer syndrome, and gastrointestinal parasitism. Some equids were found upon evaluation to have no neurologically associated diagnoses or identified deficit (*n* = 11). Another 30 patients had open diagnoses.

### Comparison Group Division

Of the 113 samples, 34 (30%) were obtained from AA centesis (“AA”), 50 (44%) from AO centesis (“AO”), and 29 (26%) from LS centesis (“LS”). One LS centesis was performed under ultrasound guidance, while the rest were performed blind. All AO centeses were performed in recumbency under general anesthesia, and all AA centeses were performed standing with ultrasound guidance. Site selection was based on clinician preference. Eighty-eight samples were classified as cytologically unremarkable after microscopic review and 25 were classified as abnormal. The predominant cell type was reviewed, which revealed 13 cases where neutrophilic pleocytosis was present. The remaining 100 samples had mononuclear cells as a predominant cell type. Attempts to independently evaluate neurologically normal samples were hindered by a low number, including only 1 patient with LS centesis.

### Comparison of Cytology and Fluid Analysis Between All Samples

When comparing all samples, RBC concentration was significantly higher (*p* < 0.05) in the LS group (median = 29 RBC/μL) than in the AA and AO groups (median = 11.5 RBC/μl from AA samples and median = 3 RBC/μL from AO samples). Furthermore, TP in AA samples (median = 40.3 mg/dL) was significantly lower (*p* < 0.05) when compared to LS (median = 55.2 mg/dL) (see Table 1).

**Table 1:** Comparison of fluid analysis parameters across all samples.

**Variables**	**AO (*n* = 50)**	**AA (*n* = 34)**	**LS (*n* = 29)**
TNCC (per μL)	1 (0–3)	2 (1-4)	2 (1-4)
Protein (mg/dL)	46.5 (35.3–71.75)	40.3 (34.55–49.9)^*^	55.2 (40.8–72.9)
RBC (per μL)	3 (0–18.25)^†^	11.5 (0–136.3)^*^	29 (15-222)

*When comparing all samples, RBC concentration was significantly higher in the LS group (median = 29 RBC/μL) than in the AA and AO groups (median = 11.5 RBC/μL from AA samples and median = 3 RBC/μL from AO samples). TP in AA samples (median = 40.3 mg/dL) was significantly decreased when compared to LS (median = 55.2 mg/dL)*.

(†) *Indicates p < 0.01*.

(*) *Indicates p- < 0.05. Values are expressed as median (interquartiles)*.

### Comparison of Cytologically Unremarkable Samples

In the subset of samples classified as cytologically unremarkable (see Table 2), samples from AA and LS were significantly higher (*p* < 0.01) in TNCC (median = 2 TNCC/μL) when compared with AO samples (AO median = 1 TNCC/μL). LS were significantly higher (*p* < 0.05) in total protein (median = 54.9 mg/dL) when compared to AA (median = 37.7 mg/dL). RBC concentration was significantly higher (*p* < 0.01) in LS (median = 32 RBC/μL) when compared to the other groups (AO median = 3 RBC/μL, AA median = 4 RBC/μL).

**Table 2:** Comparison of fluid analysis parameters across samples with normal cytology.

**Variables**	**AO (*n* = 39)**	**AA (*n* = 27)**	**LS (*n* = 22)**
TNCC (per μL)	1 (0–1)	2 (1–3)^†^	2 (1–4)^†^
Protein (mg/dL)	44.5 (33.4–58.5)	37.7 (32.7–47.9)	54.9 (39.9–69.43)^*^
RBC (per μL)	3 (0–5)^†^	4 (0–18)^†^	32 (14.75–218)

When comparing cytologically unremarkable samples, AA and LS were significantly higher in TNCC (median = 2 TNCC/μL) when compared with AO (AO median = 1 TNCC/μL). Samples from LS were significantly higher (p < 0.05) in total protein (median = 54.9 mg/dL) when compared to AA (median = 37.7 mg/dL). RBC concentration was significantly higher in LS (median = 32 RBC/μL) when compared to the other groups (AO median = 3 RBC/μL, AA median = 4 RBC/μL). (^†^) Indicates a p < 0.01. (^*^) Indicates p < 0.05. Values are expressed as median (interquartiles).

### Comparison of Cytologically Abnormal Samples

When comparing cytologically abnormal samples between sites, no significant differences were identified (see Table 3).

**Table 3:** Comparison of fluid analysis parameters across samples with abnormal cytology.

**Variables**	**AO (*n* = 12)**	**AA (*n* = 6)**	**LS (*n* = 7)**
TNCC (per μL)	8 (2.25–112)	8 (2–130.5)	4 (1–16)
Protein (mg/dL)	102.7 (41.9–142)	52.65 (48–63.65)	69 (53.8–150)
RBC (per μL)	21.5 (2–1,202)	201.5 (13.5–743)	24 (17.75–1,766)

*When comparing cytologically abnormal samples, no statistically significant differences were identified. Values are expressed as median (interquartiles)*.

### Survival Data

**Figure 1:**
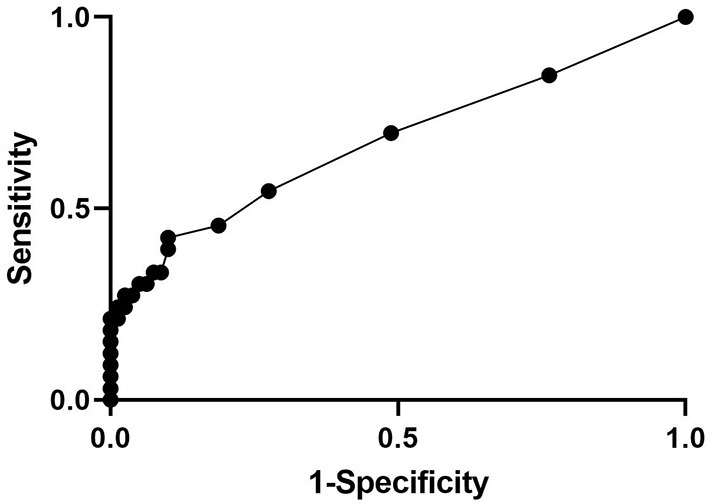
Receiver operating characteristic curve for TNCC to predict survival.

Eighty patients (71%) survived and 33 (29%) were either euthanized or died prior to discharge. CSF classified as abnormal based on fluid analysis (either morphology or quantified fluid analysis) from all 3 sites was associated (Fisher's exact test *p* = 0.0002) with non-survival (16/25 or 64% of abnormal cytology did not survive, *p* = 0.0002) when compared to normal samples (17/88 or 19% non-survival in normal samples). Non-survivors had a significantly higher (*p* < 0.01) TNCC than survivors (median non-survivors = 3 TNCC/μL, median survivors = 1 TNCC/μL) (see Table 4). The ROC curve for TNCC was evaluated to predict survival (Figure 1). The ROC curve for the TNCC had an area under the curve of 0.67 (95% CI, 0.55–0.79) and indicated that a cutoff value of 24 cells/μL maximized specificity (72%) and sensitivity (54%) to predict non-survival in all horses. Positive predictive value was 45% and negative predictive value was 78% (Figure 1).

**Table 4:** Comparison of fluid analysis parameters in survivors and non-survivors.

**Variables**	**Survivors (*n* = 80)**	**Non-survivors (*n* = 33)**
TNCC (per μL)	1 (1–3)	3 (1–19.50)^†^
Protein (mg/dL)	47.9 (37–58.7)	47 (31.23–102)
RBC (per μL)	8 (1–40.5)	14.5 (2.25–266.8)

*Non-survivors had a significantly higher TNCC than survivors (median non-survivors = 3 TNCC/μL, median survivors = 1 TNCC/μL). (^†^) Indicates p < 0.01. Values are expressed as median (interquartiles)*.

### Blood Admixture

Blood admixed samples classified as having RBC concentration ≥ 500 cells/ μL (*n* = 13) and those classified by subjective evaluation (*n* = 35) both had significantly higher (*p* < 0.05) TP, TNCC, and RBC concentration than samples classified as not blood admixed by each method (see Tables 5, 6, respectively).

**Table 5:** Comparison of fluid analysis parameters in blood admixed vs. non-blood admixed samples using an objective cutoff (>500 RBC/μL and <500 RBC/μL).

**Variables**	**>500 RBC (*n* = 13)**	**<500 RBC (*n* = 100)**
TNCC (per μL)	6 (4–48.5)^*^	1.5 (1–3)
Protein (mg/dL)	69 (6.3–150)^*^	45.9 (35.8–58.6)
RBC (per μL)	2336 (922–29,290)^†^	5 (1–27.25)

Blood admixed samples classified as having RBC concentration ≥ 500 cells/μL (n = 13) had significantly higher TP, TNCC, and RBC concentration than samples classified as not blood admixed. (^†^) Indicates p < 0.01. (^*^) Indicates p < 0.05. Values are expressed as median (range).

**Table 6:** Comparison of fluid analysis parameters in blood admixed vs. non-blood admixed samples using subjective pathologic interpretation.

**Variables**	**Blood admixture (*n* = 35)**	**No blood admixture (*n* = 78)**
TNCC (per μL)	3 (1–9)^†^	1 (0–3)
Protein (mg/dL)	55.15 (38.7–73.1)^*^	42.45 (35.7–57.4)
RBC (per μL)	161 (25–108,000)^†^	3 (0–850)

Blood admixed samples classified as those classified by subjective evaluation (n = 35) both had significantly higher TP, TNCC, and RBC concentration than samples classified as not blood admixed. (^†^) Indicates p < 0.01. (^*^) Indicates p < 0.05. Values are expressed as median (range).

## Discussion

AO centesis is traditionally performed under general anesthesia, which has inherent risks (e.g., aspiration pneumonia, recovery complications, cardiovascular instability, and adverse medication reactions) that could be compounded by neurological disease. Collection from the AO site may be preferred when attempting to assess cranial disease due to the proximity of the cerebromedullary cistern to the foramen magnum (4, 6). In addition to the inherent risks associated with general anesthesia and recovery of a potentially ataxic horse, the proximity of the AO space to the brain stem also poses a safety risk to the horse, particularly in cases with increased intracranial pressure (6). An ultrasound-guided standing approach to the AO space has been reported without adverse effects in seven horses (six neurologically normal, one neurologically abnormal) (5). However, other studies reported adverse events following this procedure when performed without ultrasound guidance including development or worsening of ataxia, collapse, hemorrhage, tonus and clonus, and temporary loss of consciousness (3, 9). There is one report of cardiovascular collapse and death of a horse following AO centesis under general anesthesia (19). Despite these adverse outcomes, it is a widely utilized procedure that anecdotally has very low rates of complication. The standing cervical CSF collection technique at the AA space provides increased access and ease of CSF collection for equids with intra-cranial and cranial cervical spinal cord disease (8).

While LS centesis may still be preferred in patients with neurolocalization of signs caudal to T2, due to caudal flow of CSF, AA puncture likely still provides a diagnostic sample in patients where minimal blood contamination and patient reaction are prioritized (8). Lumbosacral puncture may be less desirable based on clinician preference due to the safety risk assumed by the clinician performing the procedure associated with the proximity to the hind legs, the need to be elevated to obtain a dorsal approach, and explosive reactions occasionally observed in the patients (7, 8). These risks can be somewhat mitigated through appropriate sedation and restraint, such as the use of stocks, when available. Though an ultrasound-guided approach to the LS site has been described, which improved the ease of identification of landmarks, the degree of RBC concentration was unchanged when CSF collected from the LS site was compared to the CSF obtained via AA centesis (8, 9, 20). Furthermore, the importance of sampling caudal to a lesion has been found to be insignificant in other species with diffuse CNS disease due to sufficient mixing of CSF that allows representative samples to be obtained throughout the subarachnoid space (5, 7, 21).

Evaluation of CSF includes cell counts and protein quantification, morphologic evaluation, and gross characterization of the fluid (1). Reference ranges for equine CSF were predominantly established from samples obtained from the AO space although early studies did include some samples from the LS space (2). Reference intervals used at the NC State University Veterinary Teaching Hospital were determined in-house with 0–8 TNCC/μL and a TP of 0–80 mg/dL. Red blood cells are not typically present in CSF, and no standard cutoff value exists to qualify samples as blood admixed, although 500 RBC/μL has been utilized as a cutoff in other studies.

The effect of blood contamination on cell count and total protein in CSF samples has been investigated in multiple species, but with conflicting results (17, 18, 22). It has been suggested in companion animals (dogs and cats) that for every 500–750 RBC/μL increase, there is an associated increase of 1 nucleated cell/μL, and when blood contamination is present, cell counts have been corrected by subtracting nucleated cells based on this association (17, 18). However, opinions vary as to what threshold of RBC contamination truly alters the interpretation of CSF cytology (18). A retrospective study in CSF from dogs with low TNCC (≤5 cells/μL) showed that samples with greater than 500 RBC/μL had significantly higher neutrophil percentages and total protein and were significantly more likely to contain eosinophils. However, another group identified no association between degree of RBC contamination, TNCC, or protein in CSF from healthy and neurologically abnormal dogs with RBC concentration ranging from 0 to 13,230 cells/μL (18). In the population of horses described here, CSF samples that contained >500 RBC/μL and those classified subjectively on cytology as blood admixed both had significantly higher TP and TNCC. There is no standard method of classifying CSF as having blood contamination or blood admixture; thus, the same sample could be classified differently by two different laboratories, or even by different pathologists within the same institution, making interpretation of TNCC and TP elevated above reference intervals in CSF samples diagnostically challenging. Further complicating the definitions and terminology, the word “hemodilute” is frequently used to refer to these blood-contaminated samples (including in the NC State Clinical Pathology reports). However, the term “hemodilute” has been proposed to be an outdated terminology, truly meaning that blood itself is diluted. Instead, words such as “blood admixture” or “diluted with peripheral blood” have been proposed to describe these samples more accurately, leading to our terminology and classification (23). As noted in the data reported here, 35 horses were classified as being blood admixed by subjective evaluation, but only 13 when a cutoff of >500 RBC/μL was applied. Interestingly, the parameters with clinical significance did not change with either characterization. Due to the variable effect of blood contamination on CSF analytes, selecting sites where blood contamination can be minimized is the ideal practice for obtaining diagnostic samples.

Similar to previous studies, the data presented here identified significantly higher RBC concentration in samples collected from the LS site as compared to the AO and AA sites (8). As described above, higher RBC concentration can increase TNCC, which can complicate the diagnosis of certain infectious conditions that are often initially presumptively diagnosed based on the presence of mild to moderate neutrophilic pleocytosis, such as Eastern Equine Encephalitis (24). The effect of blood contamination on CSF is of additional clinical relevance when testing for EPM via serum and CSF antibody ratios, or in cases where immunodiagnostics may not be available immediately and initial therapy is guided solely by CSF cytology and fluid analysis. EPM is one of the leading causes of spinal ataxia in horses in the United States for which the most sensitive and specific ante-mortem diagnostic test is the ratio between antibody titers in the serum and CSF utilizing the surface antigen (SAG) 2,4/3 ELISA (25, 26). While results of the SAG 2,4/3 serum:CSF ratios are less likely than older tests to reflect a false positive due to a small amount of blood contamination, an effect was observed when performed on CSF samples with greater than 10,000 RBC/μL (27, 28). The same rate of RBC contamination was reported to increase the rate of false positives when utilizing indirect fluorescent antibody tests on CSF in horses that had increased serum antibody titers to *S. neurona* (29).

While elevated TNCC and abnormal cytology were associated with non-survival, when the ROC curve was calculated, data were not supportive of a strong prognostic indication of those parameters. Though a cutoff of 24 cells/μL maximized the sensitivity and specificity, sensitivity and positive predictive value remained low. An area under the curve of 0.67 is not supportive of strong predictive accuracy. This highlights that cytologic abnormalities such as a pleocytosis alone are not a strong prognostic indicator of non-survival based on these data though the association is present.

Due to the retrospective nature of this study, multiple limitations were identified. All subjects were evaluated for the presence of neurological disease. Therefore, even samples that were classified as cytologically unremarkable may have had differences from a normal population. Patient factors likely created some bias in the site from which CSF was collected. Many of the patients in which CSF was collected from the AO site were presented in recumbency or were considered unsafe to sedate for standing collection. Thus, horses with CSF collected from the AO site may have had more severe disease than those from which CSF was collected under standing sedation. Due to the difficulty in diagnosing equine neurological disease, the expense associated with diagnosis and treatment, rapid progression of clinical signs, or incomplete medical records, many patients did not have final diagnoses, which precluded evaluation of specific disease types. Each disease process may influence CSF in different ways, and therefore, the dataset available is influenced by those diseases represented in the patient population. As mentioned above, there is no standardized cutoff for classifying samples as blood admixed. While the data were analyzed using both the classification from the cytologic report and the common cutoff of ≥500 RBC/μL, the same findings may be classified differently at different institutions and thus must be interpreted with caution. While protocol has remained standard at the diagnostic lab throughout the timeframe published, the analyzers may have varied throughout that timeframe. Furthermore, reference ranges developed from the AO site were used to classify CSF as normal or abnormal, which our data suggest may not be appropriate. While this is common practice in most laboratories, reference ranges for CSF collected from the AA site in normal horses have not yet been established. A final consideration is that incomplete medical records led to the exclusion of 14 subjects from analysis due to missing data, which further decreased case numbers.

In summary, the cytologic analyses of CSF from the AO, AA, and LS sites showed significantly higher RBC concentrations in samples from the LS site. There were also differences in total protein (when comparing all samples) and TNCC (cytologically unremarkable samples). Differences in these sites in TP and TNCC have been identified in other species, and have been attributed to possible variations in permeability of the subarachnoid space or variable rates of cellular lysis (12). However, despite these changes being statistically significant differences, an important note is that all median values fell within accepted reference ranges for equine CSF, making it unlikely that these differences are clinically significant. However, mild differences in cell count and TP within those reference intervals may be relevant and warrant further investigation in a larger number of horses without evidence of neurological disease. Furthermore, greater RBC contamination from LS centesis may indicate that an alternate sampling site should be considered when evaluating comparative diagnostics, such as serum:CSF antibody ratios to test for *Sarcocystis neurona*. The data presented here suggest that differences in CSF TP and TNCC collected from 3 sites are unlikely to be of major clinical significance, but comparison of CSF from all 3 sites in normal horses is still warranted.

## Data Availability Statement

The raw data supporting the conclusions of this article will be made available by the authors, without undue reservation.

## Ethics Statement

Ethical review and approval was not required for the animal study because retrospective review of data collected as part of clinical procedures undergone independent of research conducted. Written informed consent was obtained from the owners for the participation of their animals in this study.

## Author Contributions

KH-W and KY performed initial exclusion and qualification of data. KY, KH-W, and KD all contributed to the manuscript preparation article. KD performed the statistical analysis. All authors contributed to the article and approved the submitted version.

## Funding

North Carolina State University College of Veterinary Medicine provided the publication fees.

## Conflict of Interest

The authors declare that the research was conducted in the absence of any commercial or financial relationships that could be construed as a potential conflict of interest.

## Publisher's Note

All claims expressed in this article are solely those of the authors and do not necessarily represent those of their affiliated organizations, or those of the publisher, the editors and the reviewers. Any product that may be evaluated in this article, or claim that may be made by its manufacturer, is not guaranteed or endorsed by the publisher.

## Abbreviations

AA: Atlantoaxial
AO: Atlanto-occipital
CSF: Cerebrospinal fluid
LS: Lumbosacral
TNCC: Total nucleated cell count
TP: Total protein.

## References

[1] Mayhew IG. Collection of cerebrospinal fluid from the horse. Cornell Vet. (1975) 65:500–11. 1192748

[2] Beech J. Cytology of equine cerebrospinal fluid. Vet Pathol. (1983) 20:553–62. 10.1177/030098588302000507 6636463

[3] Spinelli J Holliday T Homer J. Collection of large samples of cerebrospinal fluid from horses. Lab Anim Care. (1968) 18:565–7. 4247424

[4] Pease A Behan A Bohart G. Ultrasound-guided cervical centesis to obtain cerebrospinal fluid in the standing horse. Vet Radiol Ultrasound. (2012) 53:92–5. 10.1111/j.1740-8261.2011.01855.x 21831242

[5] Depecker M Bizon-Mercier C Couroucé-Malblanc A. Ultrasound-guided atlanto-occipital puncture for cerebrospinal fluid analysis on the standing horse. Vet Rec. (2014) 174:45. 10.1136/vr.101758 24225443

[6] Johnson PJ and Constantinescu GM. Collection of cerebrospinal fluid in horses. Equine Vet Educ. (2000) 12:7–12. 10.1111/j.2042-3292.2000.tb01755.x

[7] Mackay RJ. Developments in ultrasound-guided thecal puncture in horses. Vet Rec. (2014) 174:43–4. 10.1136/vr.g9 24413298

[8] Chidlow H Giguère S Camus M Wells B Howerth E Berghaus R. Comparison of 2 collection methods for cerebrospinal fluid analysis from standing, sedate adult horses. J Vet Intern Med. (2020) 34:972–8. 10.1111/jvim.15702 31977116 PMC7096653

[9] Pease AP. Cerebrospinal fluid standing tap. In: Sprayberry KA, Robinson NE, editors. Robinson's Current Therapy in Equine Medicine. 7th ed. Amsterdam: Elsevier Inc. (2015). p. 366–8.

[10] Weisner B Bernhardt W. Protein fractions of lumbar, cisternal, and ventricular cerebrospinal fluid. J Neurol Sci. (1978) 37:205–14. 10.1016/0022-510X(78)90204-6 681976

[11] Bailey CS Higgins RJ. Comparison of total white blood cell count and total protein content of lumbar and cisternal cerebrospinal fluid of healthy dogs. Am J Vet Res. (1985) 46:1162–5. 4003891

[12] Lampe R Foss KD Vitale S Hague DW Barger AM. Comparison of cerebellomedullary and lumbar cerebrospinal fluid analysis in dogs with neurological disease. J Vet Intern Med. (2020) 34:838–43. 10.1111/jvim.15700 31953970 PMC7096600

[13] Mayhew IG Whitlock RH Tasker JB. Equine cerebrospinal fluid: reference values of normal horses. Am J Vet Res. (1977) 38:1271–4. 911095

[14] Andrade DGA de Cerri FM Barbosa GVM Basso RM Takahira RK Pantoja JC de F. Sequential cerebrospinal fluid sampling in horses: comparison of sampling times and two different collection sites. J Equine Vet Sci. (2022) 108:103794. 10.1016/j.jevs.2021.103794 34800797

[15] Rossdale PD Cash RSG Leadon DP Lodge B Road S W SCB. Biochemical constituents of cerebrospinal fluid in premature and full term foals. Equine Vet J. (1982) 14:134–8. 10.1111/j.2042-3306.1982.tb02367.x 7084197

[16] Mozaffari AA Samadieh H. Analysis of serum and cerebrospinal fluid in clinically normal adult miniature donkeys. N Z Vet J. (2013) 61:297–9. 10.1080/00480169.2012.757724 23444916

[17] Chrisman CL. Cerebrospinal fluid analysis. Vet Clin North Am Small Anim Pract [Internet]. (1992) 22:781–810. 10.1016/S0195-5616(92)50077-8 1641918

[18] Doyle C Solano-Gallego L. Cytologic interpretation of canine cerebrospinal fluid samples with low total nucleated cell concentration, with and without blood contamination. Vet Clin Pathol. (2009) 38:392–6. 10.1111/j.1939-165X.2009.00132.x 19392761

[19] Bennell AJ Bardell D. Asystole associated with cerebrospinal fluid collection in a 3-month-old foal under general anaesthesia. Equine Vet Educ. (2020) 33:e298–e302. 10.1111/eve.13345

[20] Aleman M Borchers A Kass PH Puchalski SM. Ultrasound-assisted collection of cerebrospinal fluid from the lumbosacral space in equids. J Am Vet Med Assoc. (2007) 230:378–84. 10.2460/javma.230.3.378 17269870

[21] Bersan E. Samples collected from the cerebellomedullary cistern of steroid-responsive meningitis arteritis in dogs. J Am Vet Med Assoc. (2019) 255:1035–8. 10.2460/javma.255.9.1035 31617801

[22] Hurtt AE Smith MO. Effect of iatrogenic blood contamination on CSF in dogs. JAVMA. (1997) 211:866–7. 9333087

[23] Bohn AA. Hemodilution: What is in a word? Vet Clin Pathol. (2021) 50:476–7. 10.1111/vcp.13103 34970754

[24] Fish EJ Bertone JJ. Cerebrospinal fluid analysis in horses and large animals. In: Seelig D, Sharkey L, Radkin MJ, editors. Veterinary Cytology. 1st ed. Hoboken, NJ: John Wiley & Sons (2020). p. 655–63.

[25] Schwarz B. Cerebrospinal fluid collection and its analysis in equine neurological disease. Equine Vet Educ. (2006) 18:243–8. 10.1111/j.2042-3292.2006.tb00456.x

[26] Reed SM Furr M Howe DK Johnson AL Mackay RJ Morrow JK. Equine protozoal myeloencephalitis: an updated consensus statement with a focus on parasite biology, diagnosis, treatment, and prevention. J Vet Intern Med. (2016) 30:491–502. 10.1111/jvim.13834 26857902 PMC4913613

[27] Reed SM Howe DK Morrow JK Graves A Yeargan MR Johnson AL. Accurate antemortem diagnosis of equine protozoal myeloencephalitis (EPM) based on detecting intrathecal antibodies against sarcocystis neurona using the SnSAG2 and SnSAG4/3 ELISAs. J Vet Intern Med. (2013) 27:1193–200. 10.1111/jvim.12158 24033423

[28] Furr M Howe D Reed S Yeargan M. Antibody coefficients for the diagnosis of equine protozoal myeloencephalitis. J Vet Intern Med. (2011) 25:138–42. 10.1111/j.1939-1676.2010.0658.x 21155894

[29] Finno CJ Packham AE Wilson WD Gardner IA Conrad PA Pusterla N. Effects of blood contamination of cerebrospinal fluid on results of indirect fluorescent antibody tests for detection of antibodies against *Sarcocystis neurona* and *Neospora hughesi*. J Vet Diagnostic Investig. (2007) 19:286–9. 10.1177/104063870701900310 17459859

[30] Young KS Hepworth-Warren KL Dembek KA. 2021 ACVIM forum research abstract program: Comparison of cerebrospinal fluid between three collection sites in adult equids with neurologic disease. J Vet Intern Med. (2021) 35:2943–3079. 10.1111/jvim.16220 34351021 PMC8692203

